# Early intraocular lens explantations: 10-year database analysis

**DOI:** 10.1186/s12886-024-03570-y

**Published:** 2024-07-22

**Authors:** Maximilian Friedrich, Hyeck-Soo Son, Oliver Hassel, Lilly Teich, Victor Aristide Augustin, Ramin Khoramnia, Gerd Uwe Auffarth, Timur Mert Yildirim

**Affiliations:** Department of Ophthalmology, The David J Apple Center for Vision Research, University of Heidelberg, Im Neuenheimer Feld 400, 69120 Heidelberg, Germany

**Keywords:** Cataract, Intraoperative, IOL exchange, Complication, Surgery, Intraocular lens

## Abstract

**Background:**

The aim of this study was to analyze the causes and characteristics of IOL explantation within the first year after primary implantation.

**Methods:**

In this retrospective, cross sectional database study, a database consisting of over 2500 IOL explants sent from 199 national and international doctors over the past 10 years was analyzed. All IOLs explanted within the first year after implantation were included in this analysis. Explants with insufficient information as well as phakic and Add-on IOLs were excluded. Main outcome measures were the reason for explantation, the time between implantation and explantation, as well as IOLs’ and patients’ characteristics. Additionally, the explanted IOLs were microscopically and histologically analyzed, as required.

**Results:**

Of all explanted IOLs from the database, 1.9% (*n* = 50) were explanted within the first year after implantation. The most frequent reasons for early IOL explantation were IOL dislocation (32%), visual intolerance (26%), opacification (20%), and intraoperative complications (16%). The time between implantation and explantation was the shortest in cases with intraoperative complications (1.5 ± 3.1 days), followed by IOL dislocation (90.9 ± 103.9 days), visual intolerance (98.3 ± 86.5 days), opacifications (253.5 ± 124.0 days) and other indications (249.7 ± 124.0 days). Calcification of hydrophilic IOLs was the main type of opacification (80%). Notably, seven IOLs required immediate intraoperative exchange due to an intraoperative crack in the optic or a torn off haptic.

**Conclusion:**

Indications for early IOL explantation were IOL dislocation, visual intolerance, opacification, and intraoperative complications. Especially intraoperative damages to the IOL and early calcification show a potential for improvement of affected IOLs and implantation systems.

**Supplementary Information:**

The online version contains supplementary material available at 10.1186/s12886-024-03570-y.

## Background

Cataract surgery with subsequent intraocular lens (IOL) implantation is one of the most common surgical procedures in the world. While IOLs are implanted to last a lifetime, in some cases the IOL needs to be exchanged to maintain or improve visual quality [1–3]. Common reasons for IOL explantations include IOL dislocation, IOL opacification as well as patient dissatisfaction [1, 4]. IOL dislocation can occur due to zonular dehiscence or blunt trauma [5]. The most common type of IOL opacification requiring IOL exchange is IOL calcification of hydrophilic IOLs which can be subdivided into homogenous and localized depending its morphological pattern [6]. Another type of opacification are glistenings that may be visible in slit lamp examination in hydrophobic IOLs but only mildly deteriorate visual quality even in very advanced stages and rarely require IOL exchange [7]. Also, refractive surprises after cataract surgery with a significant patient dissatisfaction may cause an IOL exchange [5, 8]. Rare reasons for IOL exchange are postoperative complications such as uveitis-glaucoma-hyphema syndrome, corneal decompensation or endophthalmitis [2, 4]. However, not only postoperative but also intraoperative complications may necessitate an IOL exchange. IOLs can be opacified at the moment of implantation due to crystallization on the IOL surface [9, 10] or damages to the IOL can occur during the loading or implantation process [11, 12].

A problem of IOL exchange surgery is the worse visual outcome and the increased rate of complications compared to routine cataract surgery [13–15]. Son et al. analyzed a large US American registry with a total of 46 063 IOL exchange procedures and found that 39.5% of all patients had a visual acuity worse than 0.3 logMAR (below 20/40 Snellen equivalent) one year after surgery [15]. In a study of Märker et al. 19% of patients with IOL exchange needed further surgical procedures to manage complications such as IOL dislocation and retinal detachment [3]. While there are often years between the initial surgery and IOL exchange, in some cases the IOL, which was meant to last a lifetime, needs to be exchanged within the first year after implantation [1, 16]. To minimize the risk for early IOL explantation, the causes and characteristics of such cases need to be revealed.

Therefore, the aim of this study was to analyze causes and characteristics of early IOL explantation to identify potential factors for improvement.

## Methods

In this study, a database (The David J Apple Laboratory for Ocular Pathology, Heidelberg, Germany) consisting of 2567 IOL explants from 199 national and international doctors that were send to the laboratory from 2014 to 2024 was analyzed. With each IOL the sending surgeon provided information about the patient’s characteristics, the explanted IOL, the reason for explantation and the best corrected visual acuity before and after surgery using a standardized form. This includes the number of days the IOL was inside the eye, calculated as the difference between the date of implantation and the date of explantation. If only the month of implantation was documented by the surgeon, we assumed the implantation day to be the 15th of this month. IOLs explanted within the first year after implantation were included in this analysis. IOLs with insufficient information as well as phakic and Add-on IOLs were excluded from this study. The study was performed in conformance with the tenets of the Declaration of Helsinki and adheres to all German federal and state laws. This project solely involved laboratory analyses of IOL explants. No additional procedures on humans or animals were performed. The local Ethics Committee of the University of Heidelberg approved this study under the reference number: S-372/2012. All patients gave written informed consent on the use of their anonymized data for scientific purposes.

Cases with unsatisfied refraction and/or dysphotopsias as the reason for explantation were subsumed as visual intolerance. In cases where the IOL was explanted in our clinic, we additionally obtained information about the intra- and perioperative course.

### Microscopical analysis

All IOLs were microscopically analyzed (Olympus BX50, Olympus Corp., Shinjuku, Tokio, Japan) and photographed using a camera attached to the microscope (Olympus Camedia C-7070 Wide Zoom, Olympus Corp., Shinjuku, Tokio, Japan). IOLs with opacifications were stained with 1% alizarin red solution and von Kossa to visualize calcification deposits in the IOLs, as described in a previous study [17]. IOL calcification was classified as either homogeneous or localized depending on its distribution.

### Data analysis

Main outcome measures were the cause for explantation, the time between implantation and explantation, as well as IOL and patients’ characteristics. The statistical analysis was performed with SPSS for Windows (Version 29, IBM, Armonk, New York, USA). To describe the data, we reported the mean value, standard deviation and/or range.

## Results

Of all 2567 explanted IOLs from the database, 50 lenses (1.9%) fulfilled the inclusion criteria. The characteristics of patients with early IOL explantation are shown in Table 1. In 23 of the 50 cases (46%) preoperative symptoms were documented. The most common symptom was blurred vision, followed by dysphotopsias.

**Table 1:** Characteristics of patients requiring early intraocular lens explantation

Characteristic		Number (%)
Total number of patients		50
Age [years]		
	*Mean ± standard deviation*	66.32 ± 12.41
	*Range*	19–86
Gender		
	*Female*	26 (52%)
	*Male*	24 (48%)
Eye		
	*Right*	26 (52%)
	*Left*	24 (48%)
Symptoms*		
	*Blurred Vision*	20 (87.0% of 23 cases)
	*Dysphotopsias*	9 (39.1% of 23 cases)
	*Pain*	2 (8.7% of 23 cases)

*Cases with documentation of subjective symptoms: *n* = 23 (46%)

**Fig. 1:**
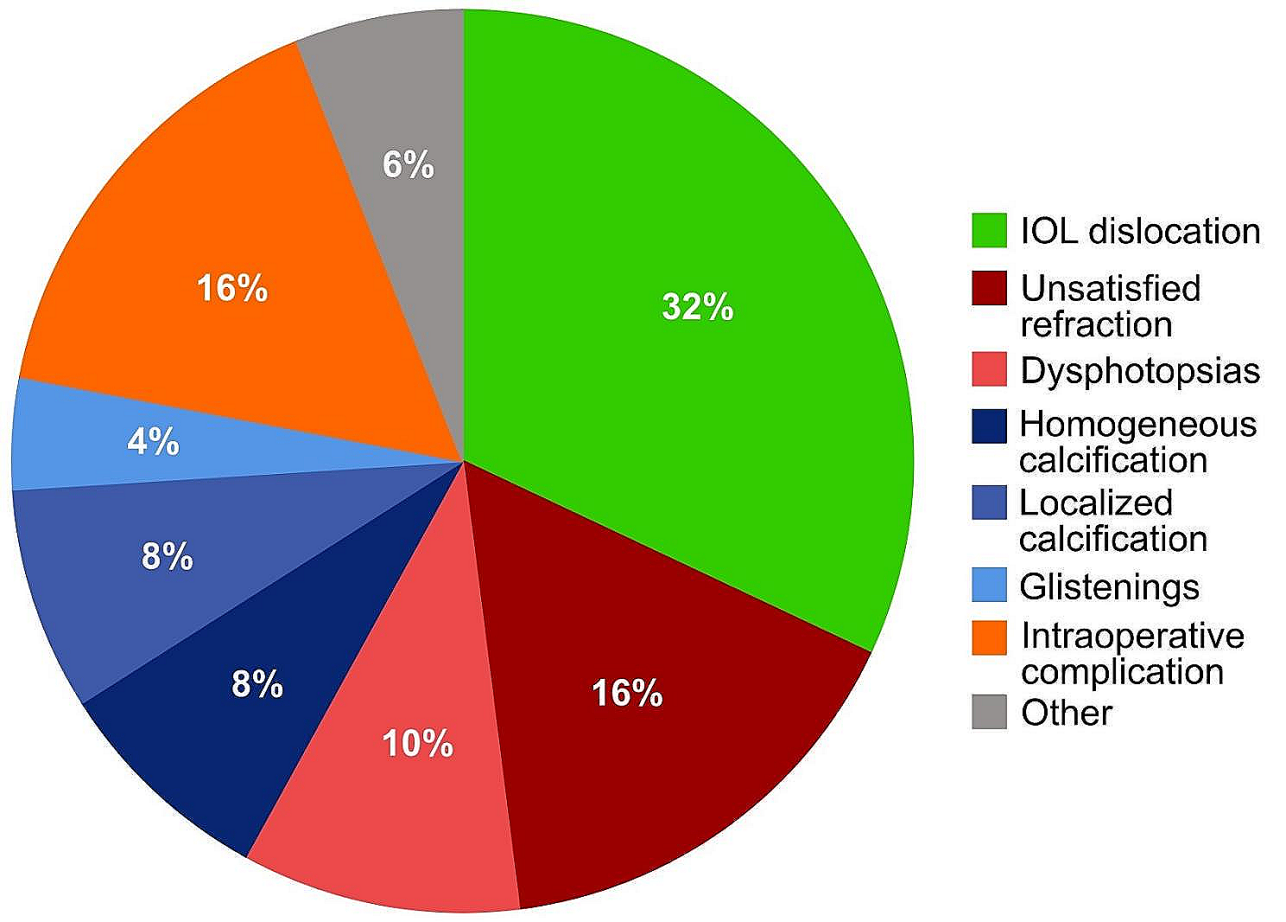
Distribution of indications for intraocular lens (IOL) explantations in the first year after IOL implantation (*n* = 50). *Green* = IOL-Dislocations. *Red* = Visual intolerance. *Blue* = Opacifications. *Orange* = Intraoperative complications. *Gray* = Other indications. Other indications: One case each with recurrent endophthalmitis, endothelial decompensation in a case with an iris-enclaved anterior chamber IOL, and one case with uveitis glaucoma hyphema syndrome

The most frequent indications for early IOL explantation were IOL dislocation (32%), visual intolerance (26%), IOL opacification (20%), and intraoperative complications (16%), as shown in Fig. 1. Other indications for early IOL explantation (6%) were recurrent endophthalmitis (*n* = 1), endothelial decompensation in a case with an iris-enclaved anterior chamber IOL (*n* = 1), and uveitis glaucoma hyphema syndrome (*n* = 1). The IOL characteristics divided by indication for IOL explantation are shown in Table 2.

**Table 2:** Characteristics of the explanted intraocular lenses (IOLs) and perioperative variables in relation to the indication for IOL explantation

Characteristic		Total	Indication for IOL explantation							
			IOL dislocation	Visual intolerance		Opacification			Intraoperative complication	Other
				Unsatisfied refraction	Dysphotopsias	Homogeneous calcification	Localized calcification	Glistenings		
Number of eyes (%)		50 (100%)	16 (32%)	8 (16%)	5 (10%)	4 (8%)	4 (8%)	2 (4%)	8 (16%)	3 (6%)
Age [years]										
	*Mean ± standard deviation*	66.3 ± 12.4	71.5 ± 7.7	61.3 ± 12.0	51.4 ± 6.9	76.0 ± 8.8	55.0 ± 24.5	70.0 ± 0.0	67.6 ± 8.5	73.3 ± 6.7
Time inside the eye [days]										
	*Mean ± standard deviation*	120.6 ± 126.5	90.9 ± 103.9	93.5 ± 109.4	106.0 ± 37.1	308.8 ± 149.9	166.5 ± 82.6	317.0 ± 0.0	1.5 ± 3.1	249.7 ± 129.9
IOL material [n]										
	*Hydrophobic acrylate*	18	10	2	4	0	0	2	0	0
	*Hydrophilic acrylate*	13	4	3	1	2	1	0	0	2
	*Hydrophilic acrylate with hydrophobic surface*	12	0	1	0	2	3	0	6	0
	*Silicone*	0	0	0	0	0	0	0	0	0
	*PMMA*	2	0	1	0	0	0	0	0	1
	*N/A*	5	2	1	0	0	0	0	2	0
IOL type [n]										
	*1-Piece*	39	8	6	5	3	4	2	8	3
	*3-Piece*	11	8	2	0	1	0	0	0	0
IOL design [n]										
	*C-Loop*	29	14	6	4	1	0	2	2	0
	*Plate haptic*	16	0	1	1	3	1	0	6	1
	*4-Point fixation*	4	2	1	0	0	0	0	0	1
	*Iris enclavation*	1	0	0	0	0	0	0	0	1
IOL fixation [n]										
	*Capsular bag*	44	14	5	5	4	4	2	8	2
	*Sulcus*	4	2	2	0	0	0	0	0	0
	*Iris*	1	0	0	0	0	0	0	0	1
	*Sclera*	1	0	1	0	0	0	0	0	0
Percentage of multifocal IOLs		14%	0%	25%	100%	0%	0%	0%	0%	0%
Preoperative CDVA [logMAR]										
	*Cases with documented* CDVA	38	16	6	5	0	2	2	5	2
	*Mean ± standard deviation*	0.61 ± 0.66	0.80 ± 0.70	0.23 ± 0.25	0.12 ± 0.11	N/A	0.40 ± 0.14	0.30 ± 0.00	1.00 ± 1.01	1.00 ± 0.14
Postoperative CDVA [logMAR]										
	*Cases with documented* CDVA	36	16	6	5	0	0	2	5	2
	*Mean ± standard deviation*	0.47 ± 0.45	0.54 ± 0.39	0.22 ± 0.12	0.10 ± 0.12	N/A	N/A	0.30 ± 0.28	0.74 ± 0.75	1.10 ± 0.28

N/A = Not available

**Fig. 2:**
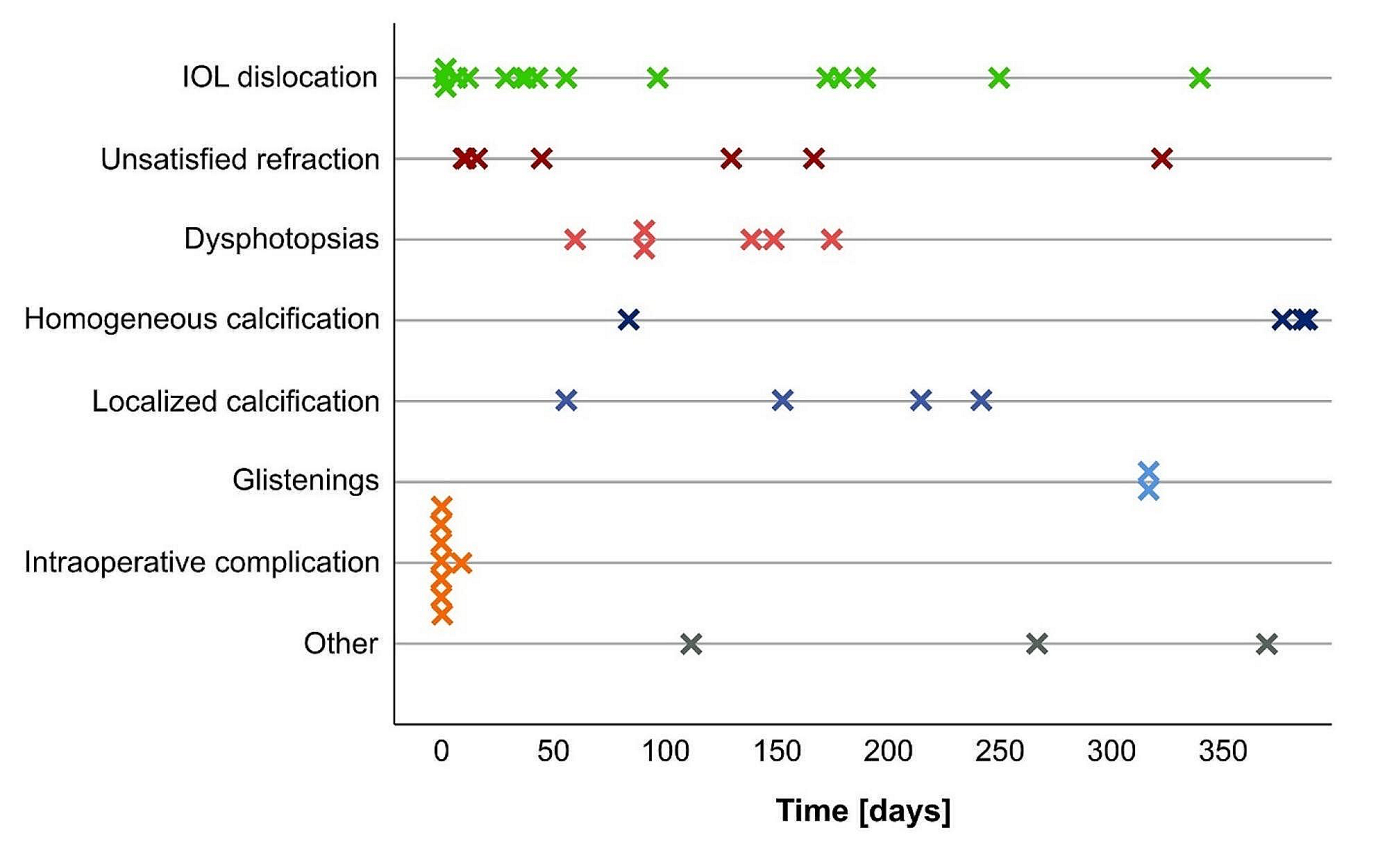
Timeline of early intraocular lens (IOL) explantations differentiated by indication of explantation. *Green* = IOL-Dislocations. *Red* = Visual intolerance. *Blue* = Opacifications. *Orange* = Intraoperative complications. *Gray* = Other indications. Other indications for early IOL explantation were recurrent endophthalmitis (*n* = 1), endothelial decompensation in a case with an iris-enclaved anterior chamber IOL (*n* = 1), and uveitis glaucoma hyphema syndrome (*n* = 1)

The time between implantation and explantation was the shortest in cases with intraoperative complications (1.5 ± 3.1 days) followed by IOL dislocation (90.9 ± 103.9 days), visual intolerance (98.3 ± 86.5 days), opacifications (253.5 ± 124.0 days) and other indications (249.7 ± 124.0 days). The individual intraocular time of each IOL is visualized on a timeline (Fig. 2).

Out of the sixteen cases with IOL dislocation, four cases had a history of vitrectomy in the affected eye (25.0%), three cases had a diagnosed pseudoexfoliation syndrome (18.8%), three cases were highly myopic (18.8%), and two cases had a dislocation related to ocular trauma (12.5%).

**Fig. 3:**
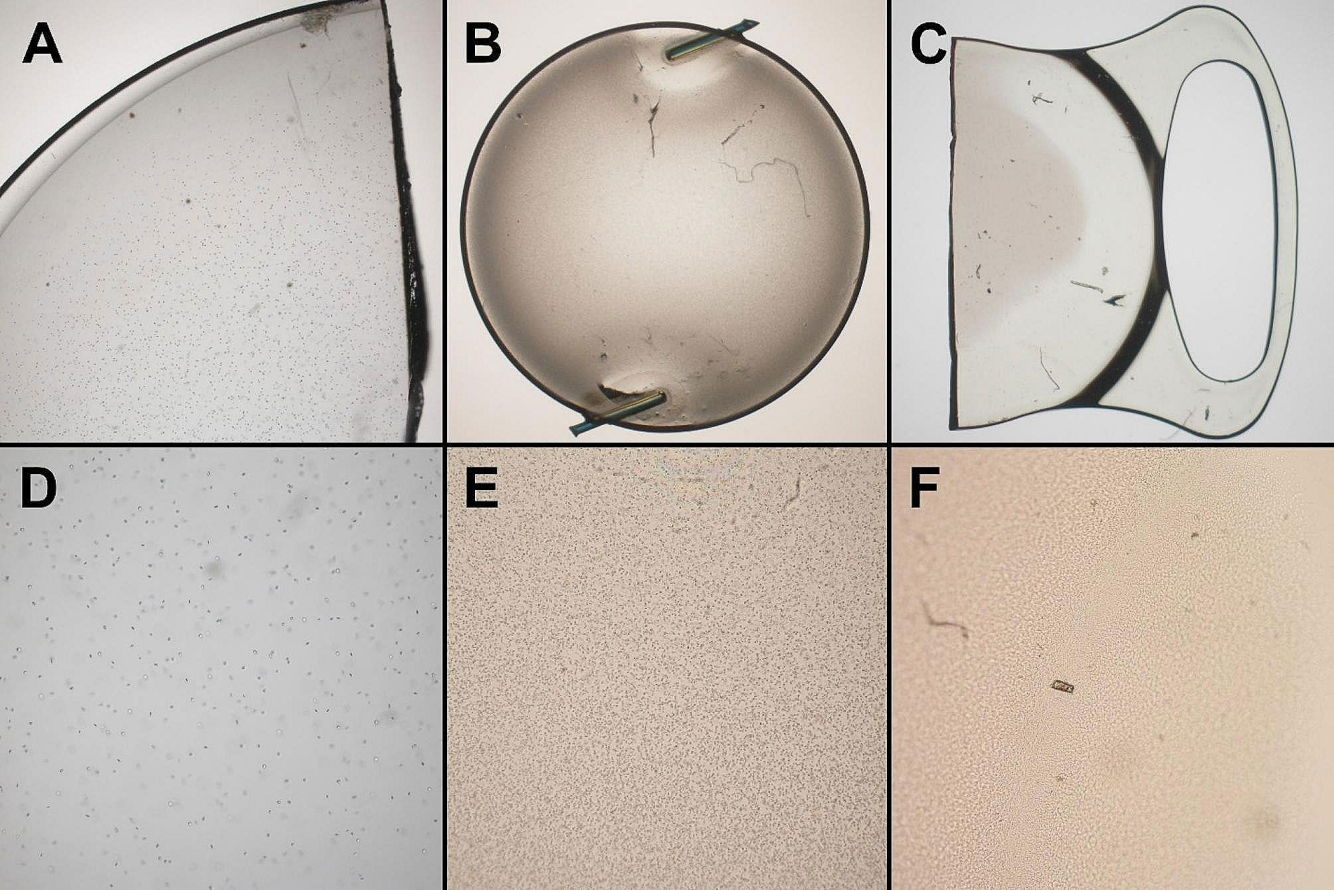
Microscopic analysis of three forms of opacifications. **A** = Glistenings in a hydrophobic IOL in 4x magnification. **B** = Homogenous calcifications in a hydrophilic IOL in 1.25x magnification. **C** = Localized calcifications in a hydrophilic IOL in 1.25x magnification. **D** = Glistenings in a hydrophobic IOL in 10x magnification. **E** = Homogenous calcifications in a hydrophilic IOL in 10x magnification. **F** = Localized calcifications in a hydrophilic IOL in 10x magnification

Homogeneous calcifications were the most frequent opacification in the explanted IOLs (40%), followed by localized calcifications (40%) and glistenings (20%). The microscopic findings are shown in Fig. 3. Calcifications were only observed in IOLs containing hydrophilic acrylate whereas glistenings were only observed IOLs containing hydrophobic acrylate. IOLs with localized calcifications were explanted predominantly earlier compared to IOLs with homogeneous calcifications. The two IOLs that were explanted due to glistenings were previously implanted in fellow eyes of the same patient.

Thirteen IOLs were explanted due to visual intolerance. The main symptom was an unsatisfying refraction followed by dysphotopsias. The preoperative best corrected distance visual acuity was 0.23 ± 0.25 logMAR in cases with unsatisfying refraction (*n* = 6) and 0.12 ± 0.11 logMAR in cases where dysphotopsias were the reason for explantation (*n* = 5). In cases with dysphotopsias, the explanted IOLs were all diffractive multifocal IOLs.

**Fig. 4:**
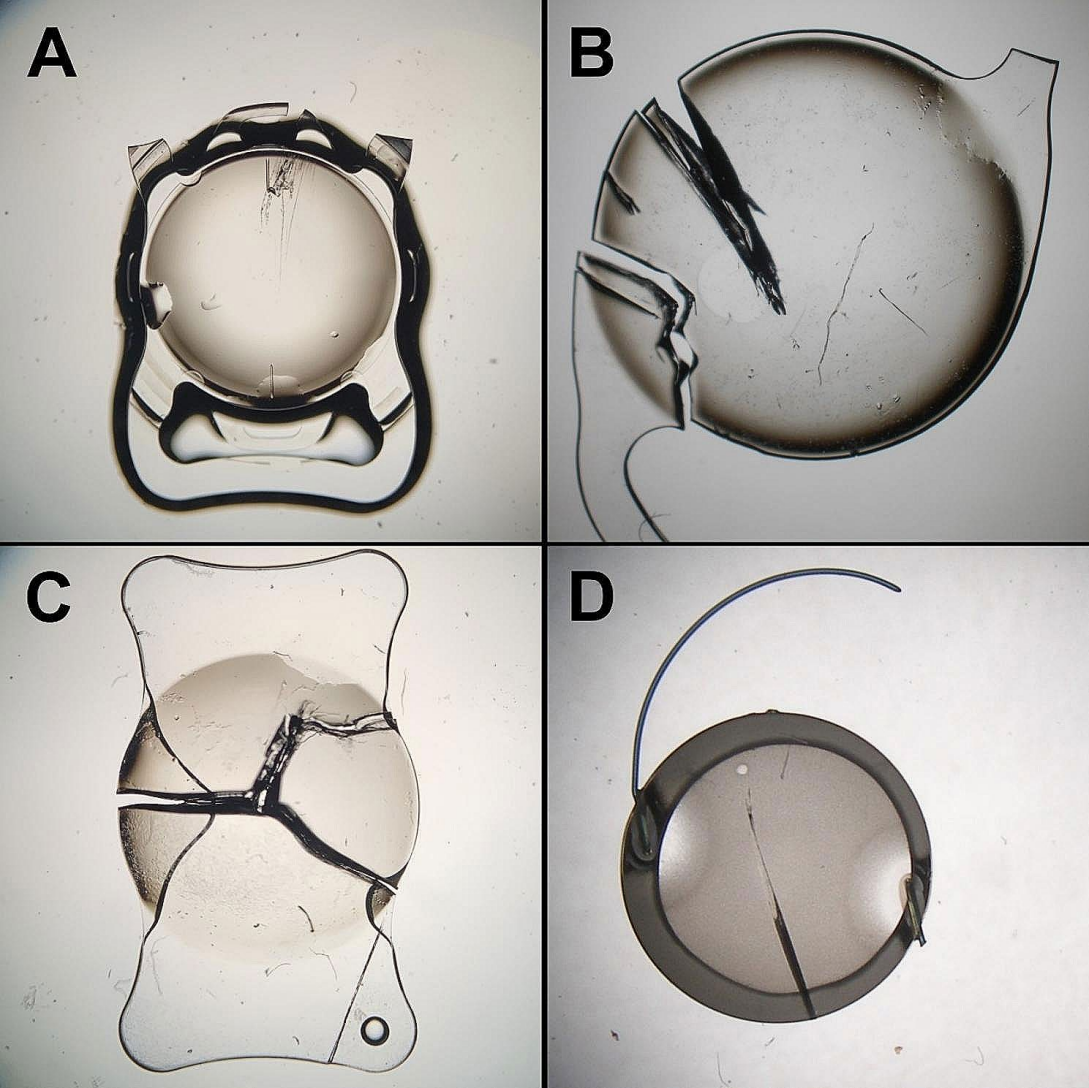
Microscopic examples of IOLs with intraoperative damage during initial implantation. **A** = Torn-off upper plate haptic. **B** = Torn-off C-loop haptic with additional crack in the optic. **C** = Crack in the optic. **D** = Crack in the optic with torn-off C-loop haptic

Out of the eight cases with intraoperative complications, four IOLs showed an isolated central crack in the IOL optic that occurred during initial implantation and was the reason for immediate subsequent explantation. An exemplary surgical video of the occurrence of this complication is shown in the Additional file 1. The remaining intraoperative complications included torn-off IOL haptics (*n* = 3) during initial implantation. Microscopic images of the damaged IOLs are shown in Fig. 4. In all cases a standard phacoemulsification technique was used with a clear corneal incision, continuous curvilinear capsulorhexis and a divide-and-conquer technique.

In one case, a native lens fragment was found on the retina nine days after externally performed cataract surgery and IOL exchange with pars plana vitrectomy was performed. This case was also classified as an intraoperative complication.

The explanted IOLs had a mean power of 20.3 diopters with a minimum of 11.5 D and a maximum of 28.0 D. Information about the IOL explantation surgery was provided in 72% (*n* = 36) of all cases. IOL explantation was predominantly performed through a sclerocorneal tunnel (52.4%). In 42.9% a clear corneal incision was used. In one case the IOL was explanted via open-sky due to simultaneous penetrating keratoplasty. The mean incision size for explantation was 5.3 ± 0.8 mm. In 91.5% of these cases, the flexible IOL was explanted through the incision in toto without intraocular fragmentation of the IOL. In the remaining cases (9.5%) intraocular IOL fragmentation was performed. A secondary IOL implantation was performed in 86.1% of all cases with available data (*n* = 36), while in the remaining five cases (13.9%) the eyes were left aphakic. Most frequently, the secondary IOL was implanted into the capsular bag (33.3%; *n* = 12), ten IOLs were fixated to the iris (27.8%), and seven IOLs were sulcus-fixated (19.4%). Only two secondary IOL implantations were sclera-fixated (5.6%).

## Discussion

This analysis included 50 IOLs that were explanted in the first year after implantation. Our study reports the frequency of early IOL explantations in relation to all IOL explantations (about 2%). The most frequent indications for an early IOL explantation were IOL dislocation, visual intolerance, IOL opacification, and intraoperative complications. While previous studies have identified IOL dislocation as the most common cause of explantation overall [5, 16], our study is the first to reveal a unique distribution of causes in the early postoperative period, highlighting the significant role of intraoperative complications. These findings identify key areas for improvement in cataract surgery, emphasizing the importance of addressing early complications to enhance patient outcomes.

In comparison to our previous study from 2020, where out of 200 explanted IOLs only two IOLs were explanted due to intraoperative complications [18], we observed an increased number of intraoperative IOL complications in the present study. In seven cases severe damage to the IOL was observed during or after IOL implantation. Notably, three incidents with an isolated full-thickness central crack in the IOL optic were observed in the same preloaded IOL model with varying diopters from one manufacturer, and three incidents with a completely torn IOL haptic were observed in another preloaded IOL model with varying diopters from another manufacturer. Potential causes for intraoperative IOL damage have been previously suggested, such as insufficient lubrication with ophthalmic viscosurgical device, manual IOL loading, IOL injector incompatibility, injector shape, IOL misconfigurations, or an overriding plunger in the IOL injector [19–23]. However, we could not identify a definite cause for the IOL damages in our study. The incidents in our study lead to immediate IOL explantation due to the potential risk for a loss of visual quality in the postoperative period. A large crack in the central optic will likely cause visual disturbances due to the crack interface hindering a satisfactory postoperative result. A completely torn IOL haptic may cause IOL decentration due to the insufficient haptic support, also leading to a suboptimal postoperative outcome with the potential need for later IOL exchange.

Nevertheless, a previous study questioned the necessity of IOL explantation in the event of IOL damage, reporting two cases where full-thickness central cracks in the IOL optic caused no significant visual disturbance postoperatively [23]. However, surgeons may critically evaluate the risk-benefit ratio in such cases.

Immediate IOL exchange might require enlarging the incision for IOL extraction or manipulating the IOL to fragment or fold it. Additionally, a back-up IOL will be required to successfully complete the surgery, resulting in an intact, uncompromised IOL and likely patient satisfaction. Yet, a larger incision and more intraocular manipulation may lead to surgically induced astigmatism and increased postoperative inflammation.

Leaving a fractured IOL in the eye could cause visual disturbances and patient dissatisfaction. While some patients may not experience postoperative visual problems, avoiding a larger corneal incision and additional IOL implantation, others might necessitate general anesthesia and face higher complication rates if IOL exchange is required later on.

Afterall, surgeons may decide individually depending on the size and position of the IOL damage as well as patient and local factors. To prevent damages in the IOL surgeons should adhere to the manufacturer’s instructions to prepare IOL implantation, and manufacturers should advance IOL technology to minimize the rate of faulty products. As these cases might be underreported, explanted damaged IOLs should be sent to an independent laboratory to systematically gather information of these cases and perform material analyses to identify production issues.

The reasons for IOL explantation vary by geographical region and period. In Spain, from 2004 to 2010, 56.3% of IOLs were explanted due to dislocation, 12.8% due to incorrect lens power, and 11.3% due to opacification [5]. A recent population study by Bothun et al. reported that IOL dislocations accounted for 72.5% of all IOL exchanges in a US American population [16]. One proposed reason for the high proportion of dislocated IOLs is the growing amount of pseudophakic patients. Combined with the suggested higher risk for IOL dislocation the longer the IOL is implanted in the eye and a longer life expectancy, the risk for an IOL dislocation grows with the pseudophakic population [8, 24]. Other risk factors for IOL dislocation are pseudoexfoliation syndrome, high myopia, retinitis pigmentosa, history of vitrectomy and trauma [13, 25, 26]. In our cohort, we found a high prevalence of these risk factors in the cases with IOL dislocation except for retinitis pigmentosa.

Over a fifth of the IOLs in our study were explanted due to visual intolerance. Despite advances in IOL power formulas, refractive surprises still occur. Additionally, patient personality significantly influences the satisfaction with the refractive outcome [27]. But also dysphotopsias can cause visual intolerance of an IOL with the need for IOL explantation. All IOLs in our study, that were explanted due to dysphotopsias, were diffractive multifocal IOLs. Multifocal IOLs are known to cause more positive dysphotopsias such as glare and halos compared to monofocal IOLs, which can lead to patient dissatisfaction [28]. While an unsatisfying refraction can be corrected by corneal refractive surgery or Add-on IOLs [29], there is currently no effective treatment for positive dysphotopsias other than IOL explantation if other causes such as a large pupil or posterior capsule opacification have been ruled out. Therefore, careful patient selection and considering alternatives like enhanced depth-of-focus (EDOF) IOLs are crucial to meet patient needs and expectations.

The mean intraocular time of IOLs explanted due to opacifications was reported to be several years [1, 5, 30]. However, a systematic review found that calcifications may occur earlier with a mean time after implantation of 14.9 ± 17.8 months [31]. Our study showed that early opacifications may decrease the visual quality and patient satisfaction leading to explantation within the first year. Homogenous and localized calcifications in hydrophilic IOLs are known to significantly increase straylight, while only localized calcifications decrease the optical quality of an IOL [32–36].

Primary calcifications, which are mostly homogeneous, predominately occur due to manufacturing issues as it has been observed in IOL models such as the Lentis LS-502-1 from Oculentis [34, 37]. Secondary calcifications, which are more often localized, are predominately caused by intraocular factors such as direct contact with air or gas due to anterior or posterior segment surgery, or are caused by systemic disease such as diabetes mellitus and blood hypertension [31, 38].

In our study, two hydrophobic acrylate IOLs were explanted by an external surgeon due to glistenings. While glistenings can be observed biomicroscopically, they only deteriorate the visual quality in advanced stages [7]. A longitudinal cases series found glistenings in the majority of patients with a hydrophobic Acrysof MA60BM IOL fifteen years after implantation, but despite increased straylight, visual function was not affected clinically relevant [39]. This questionable correlation between glistenings and visual function fuels the debate over IOL explantation for glistenings [40]. In our study, mean visual acuity was 0.30 logMAR before and after IOL exchange, supporting a restrictive approach regarding IOL exchange due to glistenings.

A previous database study by our laboratory found that IOL explantations were primarily due to opacifications (76.5%) and, to a lesser extent, dislocations (13.5%) [18]. Additionally, the proportion of IOLs explanted in the first year due to opacifications is higher compared to other studies, while the proportion of luxated IOLs is lower [5, 16]. One possible explanation is the aforementioned positive correlation between intraocular time of the IOL and the incidence of IOL dislocation. Alternatively, since the research of our laboratory is mainly known for analyzing IOL opacifications, surgeons might not send IOLs explanted due to dislocation, patient dissatisfaction, or intraoperative complications to our laboratory for examination, leading to a potential sample bias. However, a study by Goemaere et al. in Belgian hospitals showed a similar trend with IOL opacification and IOL dislocation being the most frequent reasons for IOL explantation [1].

## Conclusions

While the proportion of early explanted IOLs in comparison to the whole David J Apple Laboratory database was low (1.9%), these cases should alert surgeons and manufacturers to identify and exploit potential for improvement. IOLs are usually meant to be a lifelong implant providing excellent optical outcomes. However, we found a mean time between IOL implantation and explantation of 1.5 days in cases with intraoperative complications, around 100 days in cases with IOL dislocation and visual intolerance, and around 250 days in cases with IOL opacifications, when an IOL is explanted in the first year. Combined with a higher complication rate and worse visual outcome after IOL exchange antagonizing the safety of a routine cataract surgery, a mean age of 66 years at the time of explantation in the present study, and a mean life expectancy of more than ten years, these cases are prone to severe visual disability for a long time period. Therefore, future IOL research should focus on preventing the necessity for IOL explantations, especially in the early postoperative period.

### Electronic supplementary material

Below is the link to the electronic supplementary material.

**Supplementary Material 1: Additional file 1.** Surgical video of a central optic crack during IOL implantation in a routine cataract case

## Data Availability

The datasets used and/or analyzed during the current study are available from the corresponding author on reasonable request.

## Abbreviations

IOL: Intraocular lens
